# Oxidant–antioxidant imbalance in the serum of Myotonic Dystrophy type 1 (DM1) patients correlates with the progression of disease

**DOI:** 10.1186/1755-8166-7-S1-P88

**Published:** 2014-01-21

**Authors:** Ashok Kumar, Sarita Agarwal, Sunil Pradhan, Shubha Phadke

**Affiliations:** 1 Department of Medical Genetics, Sanjay Gandhi Post Graduate Institute of Medical Sciences, Lucknow, 226014, India; 2 Department of Neurology, Sanjay Gandhi Post Graduate Institute of Medical Sciences, Lucknow, 226014, India

## Background

Myotonic dystrophy type 1 (DM1) is the most common form of muscular dystrophy affecting adults and is due to trinucleotide sequence (CTG) in the 3′ UTR region of DMPK gene located at 19q13.3 chromosome. The increased levels of ROS/free radicals and lipid peroxides and decreased antioxidant levels play an important role in the pathogenesis of DM1.

## Aim

The intention of the present study is to assess the lipid peroxidation and antioxidant status and its association with clinical phenotypes in DM1 patients of Indian origin.

## Material and methods

Clinically diagnosed 20 DM1 patients (16 men and 4 women; median age 32.8 years±9.3, range 17–52) and 40 age and sex matched controls (32 men and 8 women; median age 31.0 years±8.6, range 16–54) were included in the study. The collected blood samples were processed for serum separation used in measurement of MDA (Cat. No. 10009055-96), SOD (Cat. No. 706002-96), GPX (Cat. No. 703102-96), GST (Cat. No. 703302-96), GSH (Tietze et al. 1969 method) and total antioxidant status or TAS (Koracevic et al. 2001 method) levels and its association with clinical phenotype were evaluated.

## Results

Analysis revealed significantly higher levels of MDA (p=0.002), SOD (p=0.006) and TAS p= 0.004) and lower level of GPX (p=0.003), GST (P<0.001) and GSH (P=0.016) in DM1 patients. A significant negative correlation of MDA level with dyspepsia and CK-MB and GST level with serum SCK, CK-MB and diabetes were observed. However, a significant positive correlation of SOD level with serum CK-MB, CK-MM and diabetes and negative correlation with facial weakness were noted. Though, GSH level had significant positive correlation with learning and writing disability, speech and languages disability yet found negative correlation with duration of the disease. The GPX and TAS showed no correlation with any clinical findings.

## Conclusion

Our data supports the pathogenic role of oxidative stress in DM1 of Indian origin and supports the opportunity to undertake clinical trials with antioxidants in this disorder.

